# Self-Inhibition Effects of Litter-Mediated Plant-Phyllosphere Feedback on Seedling Growth in Invasive and Native Congeneric Species

**DOI:** 10.3390/plants14091355

**Published:** 2025-04-30

**Authors:** Kaili Cao, Peili Shi, Xingliang Xu, Jingsheng Wang

**Affiliations:** Institute of Geographic Sciences and Natural Resources Research, Chinese Academy of Sciences, Beijing 100101, China; caokaili20@mails.ucas.ac.cn (K.C.); shipl@igsnrr.ac.cn (P.S.); xuxl@igsnrr.ac.cn (X.X.)

**Keywords:** plant-phyllosphere feedback, litter-mediated, invasion ecology, conspecific negative feedback, pathogen escape, functional traits

## Abstract

Plant-phyllosphere feedback (PPF) is an ecological process in which phyllosphere microbiota, originating from plant litter, are transmitted via aerosols and subsequently influence the growth of conspecific or heterospecific plants. However, the cross-species generality of this mechanism and its role in invasive plant success remain to be fully elucidated. This study systematically examined PPF effects using three invasive/native congeneric plant pairs from distinct families (Phytolaccaceae, Asteraceae, and Amaranthaceae) in Jiangxi Province, China. Key findings include the following: (1) Wide conspecific negative feedback across families, with four of six species exhibiting 6.2–12.7% biomass reduction under their own litter treatments (*p* < 0.05). (2) Comparable feedback intensity between invasive and native species, as indicated by average pairwise indices (invasive I = −0.05 vs. native I = −0.04; *p* = 0.15). Notably, the invasive species *Phytolacca americana* uniquely showed a positive biomass response (+7.1%), though underlying mechanisms (phytochemical or microbial) were not investigated. (3) Lack of correlation between PPF strength and plant functional traits or phylogenetic distance, as indicated by Mantel tests (*p* > 0.8), in contrast to the trait/phylogeny associations commonly observed in soil feedback systems. This study provided the first evidence of PPF universality across multiple plant families—previously documented only within Asteraceae—and highlights the potential microbial-mediated advantages in plant invasions. Future research should integrate spatiotemporal metagenomic and metabolomic approaches to decipher the dynamic pathogen/microbe networks and their phytochemical interactions.

## 1. Introduction

Plant/soil feedback (PSF) is a fundamental ecological mechanism through which plants modify soil biotic and abiotic properties, thereby influencing the performance of subsequent plant cohorts [1]. Over the past decades, PSF has been recognized as a critical driver of species coexistence, community succession, and invasion dynamics [2,3,4,5], with invasive species often escaping soil pathogens in novel ranges to gain a competitive advantage [2,6]. However, traditional PSF frameworks primarily focus on root-associated microbial interactions, largely overlooking the potential for aboveground plant/microbe interactions to generate analogous feedback via litter-mediated processes.

The phyllosphere, the microbial habitat on plant leaf surfaces, harbors taxonomically and functionally diverse communities, including bacteria, fungi, and archaea [7]. These microbes are transmitted horizontally via environmental vectors (e.g., rain, wind, and soil) or vertically through seeds [8,9], persist beyond plant senescence, colonize decaying litter [10,11,12], and influence ecosystem processes [13]. Functionally, phyllosphere microbiota range from mutualists to pathogens [7], with conspecific litter either enhancing seedling survival by suppressing pathogens [14] or facilitating host-specific disease transmission [15,16]. These contrasting roles suggest that phyllosphere-driven plant/litter interactions may operate independently of soil-mediated pathways.

Plant-phyllosphere feedback (PPF) is a recently proposed mechanism describing how litter-borne microbial communities regulate the fitness of subsequent plant cohorts [17]. Unlike PSF, PPF functions through the aerial or hydrological dispersal of litter-derived microbes, generating rapid feedback effects within days to weeks [18]. Empirical evidence from Asteraceae species demonstrates PPF ecological relevance: invasive *Ageratina adenophora* enriches pathogenic fungi in its litter to suppress conspecific seedlings [19], while native congeners exhibit similar negative feedback patterns [17,18]. These findings suggest that PPF may broadly shape plant community assembly by driving host-specific microbial accumulation.

Despite these insights, three major knowledge gaps limit the integration of PPF into ecological theory. First, PPF taxonomic generality remains untested, as studies have predominantly focused on Asteraceae. Second, the microbial escape hypothesis, a cornerstone of invasion ecology [2], has not been evaluated for phyllosphere pathogens in phylogenetically controlled systems. Third, while plant functional traits (e.g., specific leaf area) and phylogenetic relatedness predict PSF outcomes [20,21,22], their roles in shaping PPF remain unclear, despite evidence that phyllosphere microbiota are influenced by host traits and evolutionary history [23,24,25,26].

To address these gaps, this study employed a cross-family comparative approach with three key objectives: (1) to determine whether phyllosphere pathogen accumulation drives self-inhibition across plant lineages; (2) to assess whether invasive species mitigate negative PPF via escape from co-evolved litter microbes; and (3) to disentangle how host functional traits and phylogeny relatedness interact with phyllosphere microbiota to modulate feedback strength. By resolving these questions, this study advances a unified framework for understanding microbially mediated plant invasions and their role in shaping plant community dynamics.

## 2. Results

### 2.1. Phyllosphere Feedback Effects

**Figure 1:**
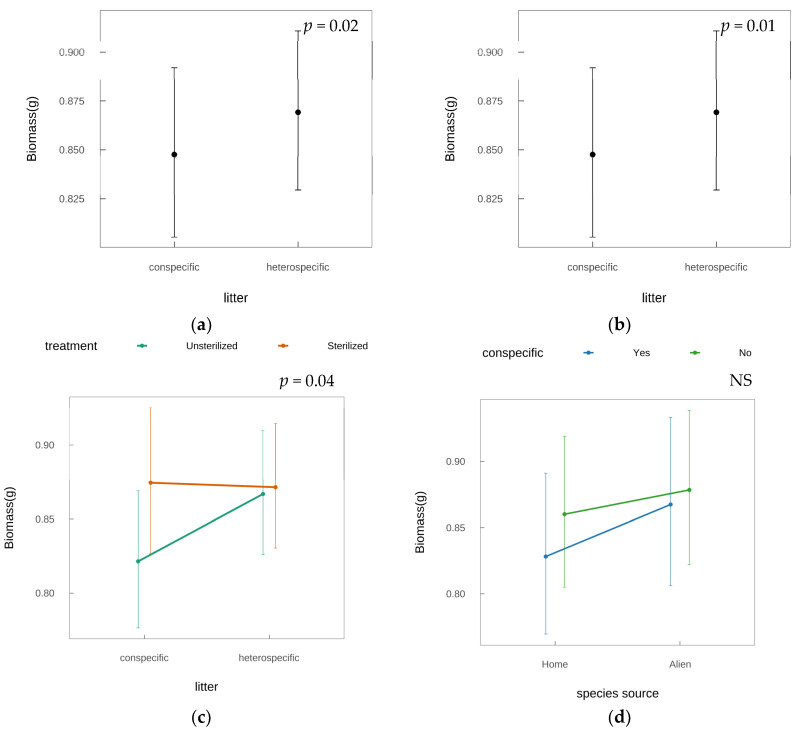
Interactive effects of sterilization treatment and litter properties on test plant biomass based on generalized linear mixed models (Gamma distribution; Table 1). (**a**) Conspecific litter main effect: biomass under conspecific vs. heterospecific litter (error bars = 95% confidence intervals; error bars = SE; n = 5). (**b**) Litter source × species source: biomass responses to native-source vs. invasive-source litter across test plant origins (error bars = SE; n = 5). (**c**) Treatment × conspecific: differential conspecific feedback strength in sterilized (orange) vs. non-sterilized (green) treatments (error bars = SE; n = 5). (**d**) Species source × conspecific: conspecific (blue = ”Yes”) vs heterospecific (green = ”No”) feedback between invasive and native plants (error bars = SE; n = 5).

GLMM analysis identified significant biomass variations associated with litter_source (invasive/native: Wald χ^2^ = 5.60, *p* = 0.02), conspecific (conspecific/heterospecific litter: Wald χ^2^ = 7.27, *p* = 0.01), and treatment (sterilized/non-sterilized: Wald χ^2^ = 6.64, *p* = 0.01; Table 1). Invasive litter treatments led to a 2.4% reduction in biomass relative to native litter (fixed-effect estimate β = −0.03, SE = 0.01, *p* = 0.02; Figure 1a). Conspecific litter reduced biomass by 5.3% relative to heterospecific treatments (β = −0.07, SE = 0.02, *p* = 0.01), aligning with Janzen–Connell negative density dependence [27,28] (Figure 1b). A significant treatment × conspecific interaction (Wald χ^2^ = 4.45, *p* = 0.04; Table 1, Figure 1c) indicated that sterilized treatments amplified conspecific litter inhibition: biomass declined by 6.1% in sterilized conspecific conditions versus non-sterilized (response ratio [sterilized/non-sterilized] = 0.939, *p* = 0.01), whereas heterospecific treatments showed no sterilization effect (response ratio = 1.00, *p* = 0.67). Invasive and native species exhibited similar responses to conspecific litter (conspecific × species_source interaction: Wald χ^2^ = 0.66, *p* = 0.42; Figure 1d), contradicting the Enemy Release Hypothesis prediction of weakened conspecific feedback in invasives.

### 2.2. Species-Specific Feedback Patterns

**Figure 2:**
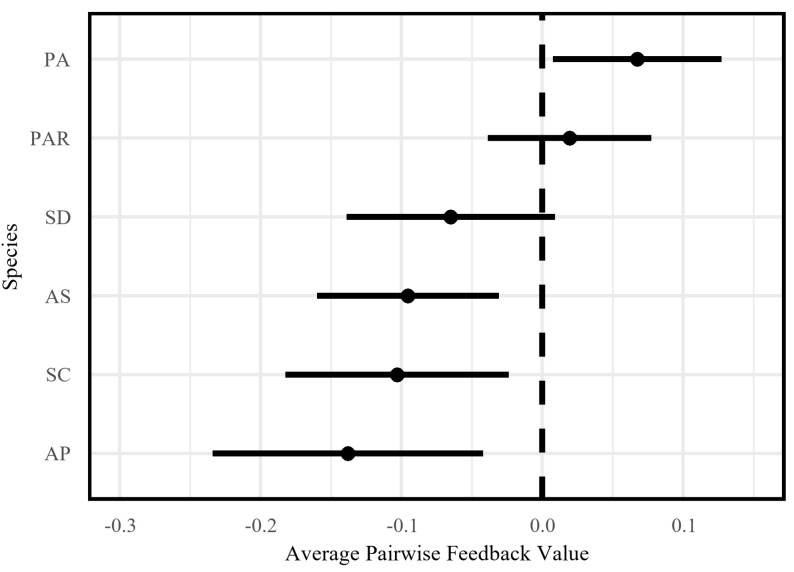
Average pairwise feedback estimates varied across plant species. The points represent species-specific mean pairwise feedback values. The error bars show 95% CIs (*t*-tests) comparing species mean pairwise feedback to neutral (zero). The black dashed line denotes neutral feedback. Species are ordered from positive to negative feedback.

**Figure 3:**
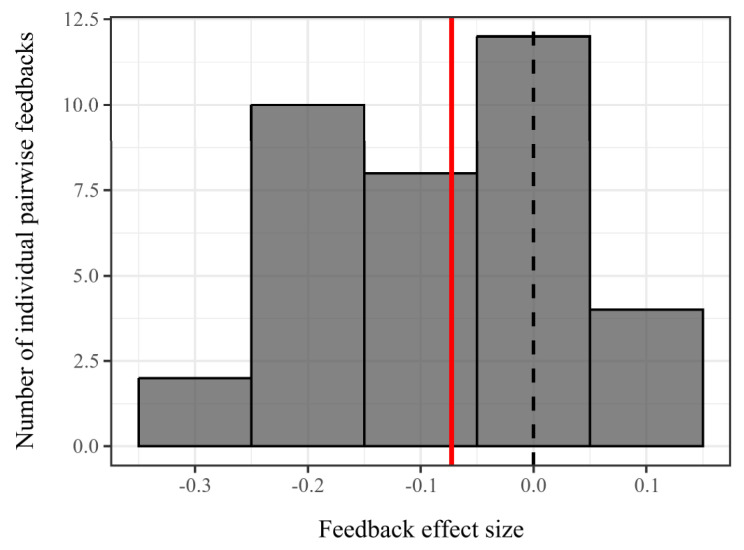
Individual pairwise feedback values between species pairs were predominantly negative (N = 30; 22 negative vs. 8 positive). The histogram displayed the distribution of all the individual pairwise feedback values. The red solid line indicates the overall mean feedback (−0.09). The black dashed line represents neutral feedback.

Of six tested species, four (AP, AS, SC, and SD) displayed negative feedback trends, with three (AP, AS, and SC) showing significant conspecific inhibition: biomass reductions of 6.2–12.7% (I = −0.138 to −0.065, *p* < 0.05; Figure 2). *Phytolacca americana* (PA) uniquely exhibited positive feedback (I = +0.067, *p* = 0.035), with conspecific litter enhancing biomass by 7.1%. No significant feedback was detected in PAR (I = +0.019, *p* = 0.406) or SD (I = −0.065, *p* = 0.071). Individual pairwise feedback (IPF) values ranged from −0.27 to +0.07, with a mean negative trend (−0.09; Figure 3). The prevalence of negative IPF values suggested that microbially mediated feedbacks predominantly generate stabilizing interactions (negative frequency dependence) between species pairs, consistent with soil feedback mechanisms that promote coexistence by suppressing conspecific dominance.

### 2.3. Functional and Phylogenetic Associations with Feedback Dynamics

**Figure 4:**
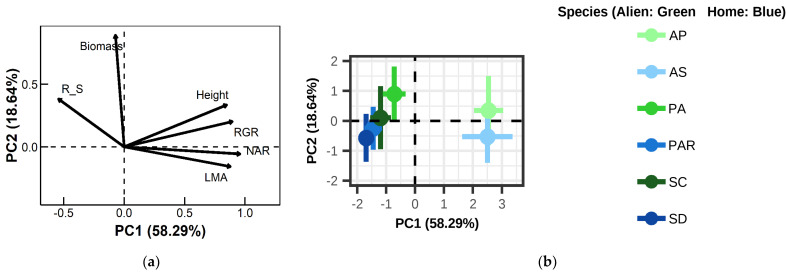
Principal component analysis (PCA) of plant functional traits categorized species along a resource acquisition/conservation continuum. Traits included total biomass (Biomass), root/shoot ratio (Root/Shoot), plant height (Height), net assimilation rate (NAR), leaf mass per area (LMA), and relative growth rate (RGR). (**a**) Arrows indicate loadings of trait vectors on the first two axes. (**b**) Colored crosses represent species means ± SD on the first two PCA axes. Color coding: alien/green; native/blue. Species abbreviations follow the methods section.

PCA of six functional traits resolved two axes explaining 77% variance (PC1: 58.29%, PC2: 18.64%; Figure 4). PC1 differentiated species with high net assimilation rate (NAR), rapid growth (RGR), tall stature, and high leaf mass per area (LMA; loadings: 0.85–0.96) from those prioritizing root allocation (root/shoot ratio [R/S]: loading = −0.54). PC2 primarily reflected total biomass (loading = 0.89). Mantel tests revealed no correlation between feedback intensity (IPF) and functional trait distance (r = −0.25, *p* = 0.83; Figure A3a) or phylogenetic distance (r = −0.12, *p* = 0.84; Figure A3b). Cubic regression detected no linkage between PC1 (life-history strategy) and average pairwise feedback (I) (r = −0.55, *p* = 0.26; Figure A5), challenging trait/phylogeny frameworks in predicting feedback dynamics [22].

## 3. Discussion

This study provides the first cross-family evidence that litter-mediated PPF regulates plant interactions, with significant interspecific variations in effect magnitude. Conspecific litter suppressed biomass by 6.2–12.7% in four of the six tested species, confirming the prevalence of negative feedback across plant lineages. These findings extend previous research on Asteraceae [17,18,19] and highlight host-specific pathogen accumulation in decomposing litter as a key driver. This mechanism is further supported by a 6.5% biomass reduction in live versus sterilized conspecific treatments (*p* = 0.01; Table 1). Notably, PPF effects (6.2–12.7%) were weaker than root-mediated PSF, which typically exceeds 15%. This discrepancy may arise from restricted microbial invasion pathways, such as stomatal penetration [7]. Additionally, aerial litter layers may act as microbial reservoirs, with hydrological dispersal mechanisms (e.g., snowmelt-mediated bacterial transport [15]) potentially amplifying PPF over space and time, necessitating further experimental validation.

Invasive and native species exhibited similar but non-significantly different feedback responses, challenging simplistic enemy release assumptions. While invasive species showed slightly weaker negative feedback (invasive I = −0.07 vs. native I = −0.11), this difference was not statistically significant (*p* = 0.39; Table 1). However, *Phytolacca americana* (PA) uniquely displayed positive feedback (I = +0.067; Figure 2), likely driven by its high foliar saponin content—a known trait in *Phytolacca* species. PA litter contained 126.5 mg g^−1^ saponins [29,30], which may suppress soil pathogens while selectively enriching arbuscular mycorrhizal fungi [31,32,33,34]. In this study, PA leaf litter was suspended in the air (without contact with soil or host plants). Its non-sterilized treatment significantly promoted seedling growth, while sterilization abolished this promotive effect and even converted it into inhibition (Figure A2b). This phenomenon suggests the potential existence of a growth-promoting mechanism independent of soil microorganisms, with the following specific hypotheses: saponins released from the litter may reach the seedling phyllosphere via volatilization or aerosol transmission, potentially enhancing host fitness by suppressing phyllosphere pathogens (e.g., fungi) or recruiting beneficial microbes (e.g., antagonistic microbes). The loss of growth promotion and shift to inhibition after autoclaving PA litter (Figure A2b) could involve multiple mechanisms: sterilization may inactivate phyllosphere-associated microbes (e.g., putative beneficial bacteria), reducing their ability to suppress pathogens or mediate nutrient transformation; alternatively, high temperature and pressure during autoclaving might degrade saponins into directly toxic derivatives or disrupt the stability of other growth-promoting compounds, such as phytohormones.

PPF likely regulates species coexistence by imposing aboveground “recruitment limitation”, where conspecific litter reduces seedling biomass by up to 12.7%—a magnitude sufficient to mitigate conspecific density dependence in natural communities. However, this mechanism operates within two ecological thresholds: (1) a trade-off between litter-driven physical facilitation (e.g., microclimate modulation; [33,35]) and microbial inhibition; (2) temporal decoupling of PPF (seasonal litter inputs) and PSF (persistent soil interactions). These observed positive feedback in PA demonstrates that certain species may circumvent these constraints by leveraging allelochemical/microbial interactions, presenting a novel invasion pathway.

Contrary to PSF studies linking feedback strength with functional traits and phylogeny [21,22], PPF showed no correlation with trait or phylogenetic distances (Mantel *p* > 0.8). This discrepancy may stem from phyllosphere-specific factors, including the following: (1) unmeasured traits (e.g., cuticle thickness and stomatal density) influencing microbial colonization [33]; (2) horizontal gene transfer enabling pathogens to bypass phylogenetic barriers [7]; (3) dynamic feedback windows during litter decomposition [34]. These findings underscore the need for revised theoretical models incorporating phyllosphere-specific drivers, such as allelochemical release kinetics, and aerial microbial dispersal.

Despite its novel contributions, this study has several limitations: (1) incomplete resolution of microbial mechanisms, particularly the dynamics between pathogens and mutualists; (2) unverified allelochemical/microbial interactions underlying the positive feedback of PA; and (3) uncertainties in scaling laboratory findings to field conditions. Although the dual-chamber system effectively isolated microbial effects (Figure A2), volatile organic compounds (VOCs) from decomposing litter may have influenced results—a limitation that could be addressed using activated carbon filtration [35]. Furthermore, the relative contributions of epiphytic versus endophytic microbiota remain unresolved. Future studies employing surface sterilization combined with tissue dissection could help disentangle their respective roles [36].

Future research should prioritize the following: (1) multi-omics integration (e.g., metabolomics and metagenomics) to elucidate tripartite allelochemical/microbial host interactions; (2) cross-scale experimental designs (e.g., litter removal gradients) to quantify the community-level impacts of PPF; and (3) climate-controlled assessments of aerial microbial dispersal efficiency under warming scenarios. By demonstrating the regulatory role of aerial litter-mediated interactions, this study challenges the conventional soil-centric PSF framework. Invasive species like PA may exploit dual ecological advantages: aerial facilitation via allelochemical-mediated microbiome engineering and below-ground enemy release. The systematic monitoring of invader litter microbiomes and allelochemical dynamics could refine invasion risk models. Ultimately, advancing our understanding of cross-habitat plant/microbe interactions—from phyllosphere to litter and rhizosphere—will facilitate a paradigm shift from reductionist perspectives to holistic systems ecology, offering actionable insights for biodiversity conservation and ecosystem management under global change pressures.

## 4. Materials and Methods

### 4.1. Study Species Selection and Phylogenetic Validation

Target species were selected based on previous surveys of invasive plants in Jiangxi Province [37] and our field investigations in Taihe County, Jiangxi Province. Three invasive plant species with invasion histories spanning at least 80 years [38]—*Solidago canadensis* (SC), *Alternanthera philoxeroides* (AP), and *Phytolacca americana* (PA)—paired with their co-occurring native congeners (*S. decurrens* (SD), *A. sessilis* (AS), and *P. acinosa* (PAR), respectively) were identified. These species were chosen for their abundance and wide distribution in the study area, with invasive/native pairs (SC-SD, AP-AS, PA-PAR) occupying overlapping ecological niches (Table A1). Taxonomic identities and life-history traits were validated via the Plants of the World Online database (POWO, www.plantsoftheworldonline.org (accessed on 1 December 2024)), while invasion status was classified according to established ecological criteria [39]. To resolve phylogenetic relationships, nuclear ribosomal ITS sequences (GenBank accession numbers in Table A2) were analyzed using maximum likelihood (ML) phylogeny in R 4.4.2 with 1000 bootstrap replicates to assess node support (Figure A4).

### 4.2. Soil Preprocessing and Substrate Standardization

Standardized substrates were prepared to ensure ecological reproducibility. Surface soil (0–20 cm depth) was collected from fallow farmland devoid of target species. After sieving (2 mm mesh) to remove roots and gravel, the soil was air-dried for physicochemical characterization: pH (potentiometric method, soil/water = 2.5:1), organic carbon (K_2_Cr_2_O_7_ oxidation–FeSO_4_ titration), total nitrogen (Kjeldahl digestion), and total phosphorus (molybdenum–antimony colorimetry) [40]. To minimize interference from native soil microbiota, background soil was mixed with autoclaved (121 °C, 30 min) quartz sand (2:1 *v*/*v*). Each pot (15 cm diameter × 20 cm height) received 0.6 L of homogenized substrate and was equilibrated under natural light for two weeks to stabilize physicochemical properties and microbial dynamics.

### 4.3. Experimental Design and Litter-Mediated Interaction Manipulation

**Figure A1:**
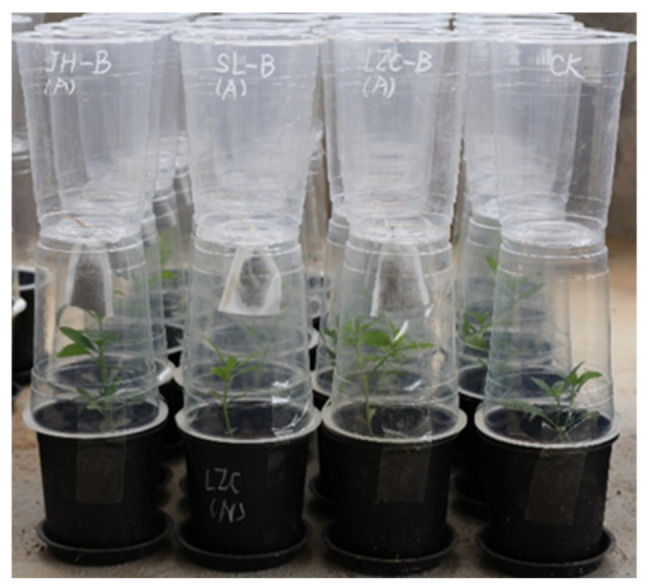
Schematic diagram of the hollow experimental setup.

**Figure A2:**
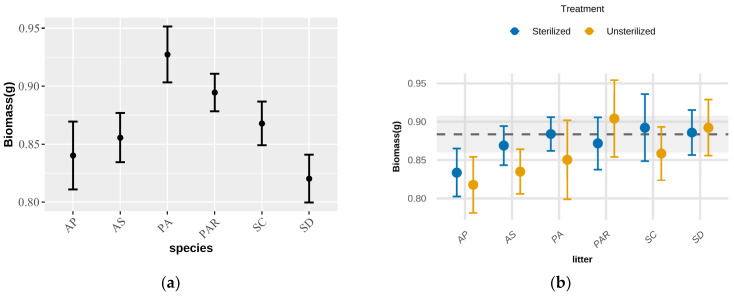
Plant biomass varied by test species (**a**) and inoculum origin (**b**). The *y*-axis represents the total plant biomass at harvest. The points indicate the mean biomass values, and th elines denote the 95% confidence intervals. The gray dashed lines and shaded regions represent the mean biomass and 95% confidence intervals for species without litter inoculation.

**Figure A3:**
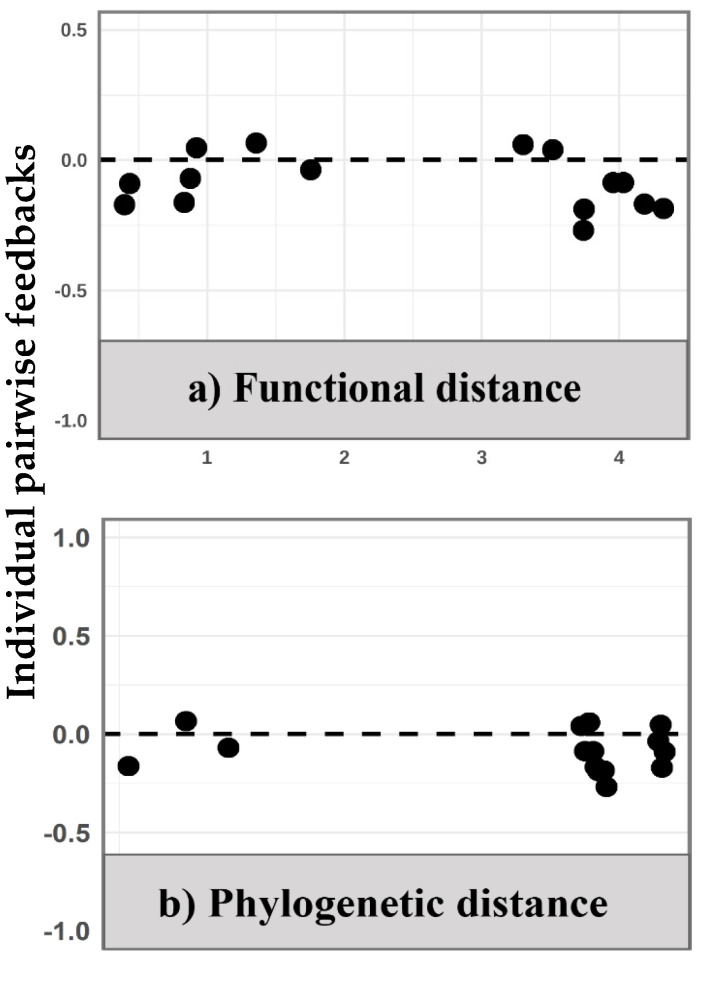
Significant correlations between individual pairwise feedback and (**a**) functional trait distance or (**b**) phylogenetic distance. The black dashed line denotes neutral feedback. Each point represents a pairwise comparison between two species (N = 30).

**Figure A4:**
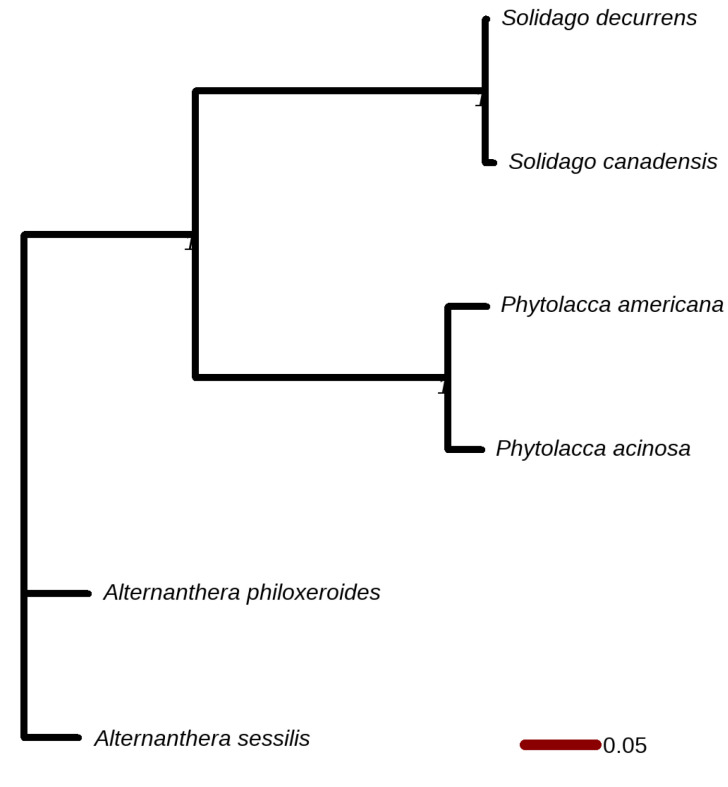
Phylogenetic tree of the six study species constructed using maximum likelihood (ML) optimization (all the nodes supported by Bootstrap values > 90).

**Figure A5:**
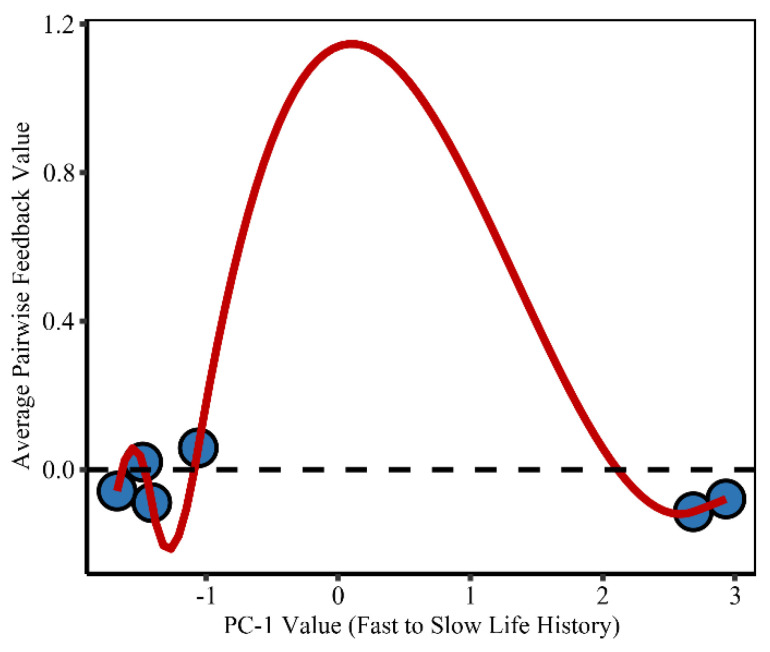
Non-significant negative trend between plant life-history strategies (PCA axis 1) and average pairwise feedback values (Pearson’s r = −0.549, *p* = 0.259; cubic polynomial regression model explained 58.3% of feedback variance, R^2^ = 0.625). PCA axis 1 represents a growth strategy gradient: positive values (PC1 > 0) correspond to “fast-strategy” species with high leaf mass allocation, strong nitrogen acquisition, and rapid growth, whereas negative values (PC1 < 0) represent “conservative-strategy” species with high root/shoot ratios. The points denote individual species (N = 6). The black dashed line indicates neutral feedback.

A custom dual-chamber cultivation system (Figure A1) was developed to separate litter/phyllosphere microbial interactions from root/soil feedback. Litter was collected from natural invasion/native habitats (26.79–26.85° N, 114.76–114.78° E), air-dried (30 °C, one week), and homogenized into 2 cm fragments. Sterilized nonwoven bags (5.5 × 7 cm) containing 3 g of litter—reflecting per-square-meter litter mass in field surveys [18]—were suspended in the upper chamber to establish spatially isolated litter layers. Three treatments were applied as follows: (1) conspecific litter interaction (intraspecific effect), (2) heterospecific litter interaction (interspecific effect), and (3) autoclaved litter (121 °C, 30 min) as abiotic control. A full factorial design (N = 330 pots) included five biological replicates for conspecific pairs, four for heterospecific pairs, and five no-litter controls per species. All the pots were randomized in a greenhouse to minimize microenvironmental heterogeneity. Consistent with prior PPF detection timelines [17,18,19], the experiment was conducted over an 8-week period—a sufficient duration to reliably capture the critical phases of PPF dynamics, as validated by the rapid microbial colonization and measurable host-specific feedback effects reported in these studies.

### 4.4. Phenotypic Measurement and Functional Trait Quantification

At harvest, phenotypic traits were measured using standardized protocols: (1) morphological metrics: plant height (digital caliper, ±0.01 cm precision) and total leaf count; (2) biomass allocation: roots, stems, and leaves were separated, weighed fresh, oven-dried at 65 °C to constant mass, and reweighed for dry mass; (3) functional traits: leaf mass per area (LMA = leaf dry mass/leaf area, with leaf area quantified via HP M1522n flatbed scanning and ImageJ 1.54g analysis), relative growth rate (RGR) based on temporal changes in plant size (height × leaf count), and net assimilation rate (NAR) calculated as the logarithmic ratio of dry mass increment to leaf area change [41]. These metrics collectively captured resource allocation strategies and growth dynamics critical for PPF interpretation.

### 4.5. Statistical Analyses

#### 4.5.1. Multiscale Drivers of Phyllosphere Feedback

GLMM was implemented in R 4.4.2 to dissect phyllosphere feedback mechanisms. Total biomass served as the response variable. The fixed effects included treatment (binary: sterilized/unsterilized), conspecific (binary: conspecific/heterospecific litter), species_source (alien/home), litter_source (alien/home), and the interactions treatment×conspecific and conspecific×species_source. The random effects accounted for interspecific variability by including species as a grouping factor. A Gamma distribution with a log-link function addressed right-skewed biomass residuals. The significance of the fixed effects was assessed via Type III Wald χ^2^ tests, with model convergence ensured by the Bobyqa optimizer (Table 1).

#### 4.5.2. Feedback Intensity Metrics

Average pairwise feedback (I)

This metric quantifies species-level net microbially mediated feedback by averaging across all the pairwise combinations. It is calculated as follows: I = $\frac{1}{N}\sum_{j=1}^{N}\;ln(\frac{B_{conspecific,j}}{B_{heterospecific,j}})$, where N denotes the total number of heterospecific pairings; and B_conspecific,j_ and B_heterospecific,j_ represent the biomass of a focal species grown in conspecific and heterospecific litter environments paired with species j, respectively. More negative values of i indicate microbially enhanced self-limitation [42,43].

Individual pairwise feedback (IPF)

For species pairs i and j, this index evaluates coexistence stability through litter/microbe interactions and is defined as follows: ${IPF}_{ij}$ = ln($\frac{B_{ii}B_{jj}}{B_{ij}B_{ji}}$), where *B_ii_* and *B_jj_* denote the biomass of species i and j in their own litter, while *B_ij_* and *B_ji_* represent their biomass in heterospecific litter. IPF_ij_ < 0 corresponds to stabilizing negative frequency dependence, facilitating coexistence [18,22].

#### 4.5.3. Trait/Phylogeny Relationships

Six functional traits (height, total biomass, LMA, root/shoot ratio [R/S], RGR, NAR) underwent principal component analysis (PCA), extracting PC1 (58.29% variance) and PC2 (18.64% variance) to construct functional trait distance matrices. Phylogenetic distances were derived from ML trees (bootstrap support >90%). Mantel tests assessed correlations between trait/phylogenetic distances and IPF, while cubic polynomial regression modeled PC1 (representing life-history strategy gradients) against feedback intensity (I) [18,42] (Bauer et al., 2015; Zaret et al., 2021).

## 5. Conclusions

This study establishes PPF as a key mechanism regulating interspecific interactions, driven by airborne microbial reservoirs in decomposing litter. Experimental evidence confirmed conspecific negative feedback in four species: *S. canadensis*, *S. decurrens*, *A. philoxeroides*, and *A. sessilis*. In these species, litter-borne phyllosphere microbes (e.g., pathogenic fungi) suppressed seedling growth via aerial transmission. In contrast, PA generated positive feedback (I = 0.067 *p* = 0.035), likely mediated by high foliar saponin content. The 6.5% biomass recovery in sterilized conspecific treatments (Δ = 6.5%, *p* = 0.01; Table 1) directly supports host-specific pathogen accumulation as a primary mechanism.

From a community assembly perspective, PPF may regulate spatial population dynamics through aerial “recruitment limitation”. However, its relative importance compared to root-mediated PSF requires multi-interface experimentation. The lack of correlation between PPF and functional traits/phylogeny (R^2^ < 0.3, *p* > 0.1) underscores the distinct nature of phyllosphere interactions, likely shaped by leaf structural traits and microbial adaptability rather than traditional trait-based expectations.

Future research should integrate metagenomics and cross-scale manipulative experiments to unravel the hologenomic regulatory networks governing phyllosphere microbiota. Such insights will refine our understanding of coevolutionary processes and inform biodiversity conservation and invasion management strategies. Ultimately, this study advances a shift from a “soil-centric” to a “whole-habitat interaction” perspective, emphasizing the need for integrative approaches to plant/microbe ecology in the face of global environmental change.

## Figures and Tables

**Table 1:** Results of generalized linear mixed models (Gamma distribution) testing phyllosphere feedback effects. Fixed effects: treatment (sterilized/unsterilized), conspecific (conspecific/heterospecific litter), test species source (invasive/native), litter source (invasive/native), and interactions. Random effect: species (variance < 0.01). Significance assessed via Type III Wald χ^2^ tests; * *p* < 0.05. Model converged using Bobyqa optimizer.

	Chisq	D.F.	F.*p*-Value	Variance
Fixed effects				
Intercept	17.22	1	<0.01 *	-
treatment	6.64	1	0.01 *	-
conspecific	7.27	1	0.01 *	-
species_source	0.75	1	0.39	-
litter_source	5.60	1	0.02 *	-
treatment×conspecific	4.45	1	0.04 *	-
conspecific×species source	0.66	1	0.42	
Random effects				
species	-	-	-	<0.01
Residual	-	-	-	0.01

## Data Availability

Data are contained within the article.

## Abbreviations

The following abbreviations are used in this manuscript:

GLMM	generalized linear mixed model
PPF	plant-phyllosphere feedback

## Appendix A

**Table A1:** Test species information.

Latin Name	Abbreviation	Life History	Family	Origin
*Solidago canadensis*	SC	Perennial	Asteraceae	Exotic
*Alternanthera philoxeroides*	AP	Perennial	Amaranthaceae	Exotic
*Phytolacca americana*	PA	Perennial	Phytolaccaceae	Exotic
*Solidago decurrens*	SD	Perennial	Asteraceae	Native
*Alternanthera sessilis*	AS	Perennial	Amaranthaceae	Native
*Phytolacca acinosa*	PAR	Perennial	Phytolaccaceae	Native

**Table A2:** ITS GenBank accession numbers and metadata used for phylogenetic tree construction.

Species	Accession Number	Author Citation	Base Pair Length
*Alternanthera philoxeroides*	KY968872.1	Xu, S.Z., Jin, X.H. and Li, Z.Y. [44]	715
*Alternanthera sessilis*	EU497240.1	Heenan, P.B., et al. [45]	731
*Phytolacca acinosa*	MK602343.1	Grosjean, N. and Blaudez, D	703
*Phytolacca americana*	MW843828.1	Wang, J.	716
*Solidago canadensis*	HQ142591.1	Laureto, P.J. and Barkman, T.J. [46]	811
*Solidago decurrens*	MN947289.1	Lin, Q.	685
